# The importance of meropenem resistance, rather than imipenem resistance, in defining carbapenem-resistant *Enterobacterales* for public health surveillance: an analysis of national population-based surveillance

**DOI:** 10.1186/s12879-024-09107-4

**Published:** 2024-02-15

**Authors:** Chiaki Ikenoue, Mari Matsui, Yuba Inamine, Daisuke Yoneoka, Motoyuki Sugai, Satowa Suzuki, the Antimicrobial-Resistant Bacteria Research Group of Public Health Institutes (AMR-RG-PHI), Mari Matsui, Satowa Suzuki, Yohei Takahashi, Nozomi Kamitaka, Shiho Takahashi, Nami Kanno, Takuya Ishi, Ryo Shimada, Hiroko Takahashi, Mayumi Ogawa, Koji Kikuchi, Hiroyuki Ueno, Kentaro Tomari, Junko Yoshihara, Naoshi Ando, Takako Katakura, Yuko Matsumoto, Yoko Anzawa, Satoko Haruna, Mikako Hosoya, Masanori Watahiki, Mika Shiroza, Koji Yokoyama, Makiko Noda, Ayako Furuta, Ryuji Kawahara, Kaoru Umeda, Takahiro Yamaguchi, Noriko Nakanishi, Kumiko Kuroda, Etsuko Saito, Yumiko Inoue, Yuta Kawakami, Tatsuaki Aota, Kanako Masuda, Hitoshi Ohtsuka, Chiemi Fukuda, Kazumi Seki, Yoko Iwashita, Yukiko Asano, Yuka Fukuguchi, Emi Arikawa, Rika Maeda, Tsuyoshi Kudeken

**Affiliations:** 1 Field Epidemiology Training Program, Infectious Diseases Surveillance Center, National Institute of Infectious Diseases, Tokyo, Japan; 2 Center for Field Epidemic Intelligence, Research, and Professional Development, National Institute of Infectious Diseases, Tokyo, Japan; 3 Antimicrobial Resistance Research Center, National Institute of Infectious Diseases, Tokyo, Japan; 4 Center for Surveillance, Immunization, and Epidemiologic Research, National Institute of Infectious Diseases, Tokyo, Japan

**Keywords:** Carbapenem-resistant *Enterobacterales*, Carbapenemase-producing *Enterobacterales*, Surveillance definition, Meropenem, Imipenem, IMP-type metallo-β-lactamase, Multidrug resistance

## Abstract

**Background:**

In Japan, carbapenem-resistant *Enterobacterales* (CRE) infections were incorporated into the National Epidemiological Surveillance of Infectious Diseases (NESID) in 2014, necessitating mandatory reporting of all CRE infections cases. Subsequently, pathogen surveillance was initiated in 2017, which involved the collection and analysis of CRE isolates from reported cases to assess carbapenemase gene possession. In this surveillance, CRE is defined as (i) minimum inhibitory concentration (MIC) of meropenem ≥2 mg/L (MEPM criteria) or (ii) MIC of imipenem ≥2 mg/L and MIC of cefmetazole ≥64 mg/L (IPM criteria). This study examined whether the current definition of CRE surveillance captures cases with a clinical and public health burden.

**Methods:**

CRE isolates from reported cases were collected from the public health laboratories of local governments, which are responsible for pathogen surveillance. Antimicrobial susceptibility tests were conducted on these isolates to assess compliance with the NESID CRE definition. The NESID data between April 2017 and March 2018 were obtained and analyzed using antimicrobial susceptibility test results.

**Results:**

In total, 1681 CRE cases were identified during the study period, and pathogen surveillance data were available for 740 (44.0%) cases. *Klebsiella aerogenes* and *Enterobacter cloacae* complex were the dominant species, followed by *Klebsiella pneumoniae* and *Escherichia coli*. The rate of carbapenemase gene positivity was 26.5% (196/740), and 93.4% (183/196) of these isolates were of the IMP type. Meanwhile, 315 isolates were subjected to antimicrobial susceptibility testing. Among them, 169 (53.7%) fulfilled only the IPM criteria (IPM criteria-only group) which were susceptible to meropenem, while 146 (46.3%) fulfilled the MEPM criteria (MEPM criteria group). The IPM criteria-only group and MEPM criteria group significantly differed in terms of carbapenemase gene positivity (0% vs. 67.8%), multidrug resistance rates (1.2% vs. 65.8%), and mortality rates (1.8% vs 6.9%).

**Conclusion:**

The identification of CRE cases based solely on imipenem resistance has had a limited impact on clinical management. Emphasizing resistance to meropenem is crucial in defining CRE, which pose both clinical and public health burden. This emphasis will enable the efficient allocation of limited health and public health resources and preservation of newly developed antimicrobials.

**Supplementary Information:**

The online version contains supplementary material available at 10.1186/s12879-024-09107-4.

## Background

Carbapenem-resistant *Enterobacterales* (CRE) have been represents a global public health concern, and the importance of surveillance is well recognised [1–3]. Furthermore, carbapenemase-producing *Enterobacterales* (CPE) have been prioritised as the target in CRE surveillance because of their potential to exhibit multidrug resistance and cause outbreaks in healthcare settings [1, 4, 5]. However, practical application of the definition of CRE for surveillance based on the antimicrobial susceptibility test (AST) in clinical diagnostic laboratories is cumbersome because the minimum inhibitory concentrations (MICs) of carbapenems for CPE might be below the clinical breakpoints, *Enterobacterales* consists of diverse bacteria species, and their mechanisms of carbapenem resistance are complex and inconsistent.

In Japan, patient-based CRE case surveillance was included in the National Epidemiological Surveillance of Infectious Diseases (NESID) in 2014, followed by CRE pathogen surveillance in 2017 [6]. In this national surveillance, CRE was defined as follows:

(i) MIC of meropenem ≥2 mg/L (MEPM criteria) or
(ii) MIC of imipenem ≥2 mg/L and cefmetazole ≥64 mg/L (IPM criteria).

The characteristics of this Japanese definition of CRE is a low MIC cutoff (meropenem/imipenem MIC ≥2 mg/L) and the combined usage of imipenem resistance and cefmetazole resistance (MIC ≥64 mg/L).

In establishing these criteria, numerous discussions occurred regarding the IPM criteria. It was acknowledged that imipenem was not recommended for detecting CPE because of its overlapping MIC distribution among wild-type isolates and carbapenemase producers, particularly in several species of *Enterobacterales* [7, 8]. However, imipenem was inevitably included in the CRE definition because in Japanese clinical settings in 2014, carbapenem susceptibility was primarily assessed using imipenem rather than meropenem. However, a low MIC cutoff for carbapenems was deemed necessary to enhance the sensitivity of CPE detection. The reason for combining cefmetazole resistance with imipenem resistance was to exclude intrinsic imipenem non-susceptible *Proteus mirabilis* and CTX-M-type extended-spectrum β-lactamase (ESBL) producers, which are endemic to Japan [9, 10].

Imipenem resistance is usually included in the definition of CRE in other countries. The current definition provided by the Centers for Disease Control and Prevention of United States (US-CDC) for CRE is *Enterobacterales* that are resistant to any carbapenem (i.e., MIC of ≥4 mg/L for doripenem, meropenem, or imipenem or ≥ 2 mg/L for ertapenem) or documented carbapenemase production [11]. The European Antimicrobial Resistance Surveillance Network has focused on carbapenem resistance using imipenem or meropenem, as well as multidrug resistance (defined as resistance to a combination of fluoroquinolones, third-generation cephalosporins, and aminoglycoside) in *Escherichia coli* and *Klebsiella pneumoniae* [12].

The CRE definition in national surveillance has a substantial impact on clinical management, infection control policy, and clinical research. However, since the introduction of Japanese CRE surveillance, no study has evaluated whether the IPM and MEPM criteria facilitate the effective detection of CPE and identification of CRE cases that truly pose a clinical and public health burden.

## Methods

This population-based observational study aimed to assess the current CRE definition used in Japan by analyzing data collected between April 2017 and March 2018 by national case and pathogen surveillance and the antimicrobial susceptibilities of isolates obtained from reported CRE cases.

### National surveillance data of cases of CRE infections in Japan

Physicians must report all diagnosed CRE cases to public health centres within 7 days of diagnosis. The definition of CRE used in this surveillance program was as described in the Background. CRE infection was reported if CRE was isolated from aseptic clinical specimens or from non-aseptic clinical specimens, followed by clinical confirmation that the isolated CRE was the causative infectious pathogen.

Information collected in the surveillance reporting form includes patient demographics, type of infection, CRE species, clinical specimen, date of diagnosis, and date of death, if applicable. We considered all cases whose date of death was reported as fatal within 7 days of diagnosis, to analyse the all-cause 7-day mortality.

This study analyzed the data of CRE cases diagnosed between April 1, 2017 and March 31, 2018, and the data were obtained from the NESID system on October 10, 2018. The per capita incidence of CRE infection was calculated using national statistics obtained from the Statistics Bureau of Japan. The estimated total population of Japan was 126,706,000 as of October 1, 2017.

### National pathogen surveillance data for CRE

In March 2017, pathogen surveillance for CRE was initiated as part of the NESID program in which the results of carbapenemase gene investigations have been registered. The Public Health Institute (PHI) of each local government conducted genotypic analysis of the CRE isolates from the reported CRE cases, examining the major carbapenemase genes (*bla*_IMP_, *bla*_KPC_, *bla*_NDM_, and *bla*_OXA-48-like_) using polymerase chain reaction and conducting phenotypic tests for carbapenemase production using metallo-β-lactamase inhibitors (sodium mercapto-acetic acid or EDTA) and a KPC-type carbapenemase inhibitor (boronic acid). All PHI personnel in charge of these analysis were trained at the National Institute of Infectious Diseases (NIID) for standardized surveillance. In this study, pathogen data were obtained from the NESID pathogen surveillance system on October 23, 2018 and integrated with the data of CRE cases.

### Antimicrobial susceptibility

Because AST data are not included neither in CRE case surveillance nor pathogen surveillance, isolates registered in the pathogen surveillance from the PHIs were collected in this study, and their MICs were determined for ten types of antimicrobials (flomoxef, ceftriaxone, cefepime, piperacillin–tazobactam, aztreonam, imipenem, meropenem, levofloxacin, amikacin, and tigecycline) using the MicroScan Neg MIC EN 2 J panel (Beckman Coulter, Brea, CA, USA) at NIID. Susceptibility breakpoints for each antimicrobial were determined with reference to the 26th edition of the Clinical and Laboratory Standards Institute (CLSI) M100 [13]. The breakpoints for flomoxef and tigecycline, which are not included in the CLSI criteria, were defined as follows: flomoxef, ≤8 mg/L for susceptible and > 32 mg/L for resistant; and tigecycline, ≤0.5 mg/L for susceptible and > 0.5 mg/L for resistant.

### Statistical analysis

The Wilcoxon rank-sum test was used to analyze statistically significant differences in continuous variables, whereas categorical variables were analyzed using Fisher’s exact test.

The statistical association between the variables of interest and all-cause 7-day mortality was evaluated using a logistic regression approach. Statistically significant risk factors identified by univariate logistic regression were further evaluated using multivariable logistic regression.

Statistical tests were two-sided, and *p* < 0.05 denoted statistical significance. Hommel-type corrections were conducted for multiple tests when necessary to appropriately identify the statistical association between outcomes and mortality. In addition, when a clear association was observed with mortality prior to the tests, the significance was subjected to one-sided testing with a threshold of *p* < 0.05. Statistical analyses were performed using JMP 13.2.1 (SAS Institute Inc., Cary, NC, USA) and RStudio (2021.09.0 + 3519).

## Results

### Description of CRE cases in Japan

In total, 1681 cases of CRE infection were reported across all 47 prefectures of Japan between April 2017 and March 2018; thus, the annual incidence was 1.33 cases per 100,000 population.

Pathogen surveillance data were available for 740 (44.0%) of the 1681 reported cases. Carbapenemase gene-positive *Enterobacterales* (CgPE) infection was confirmed in 196 cases (26.5%, Table 1). The clinical features of CgPE and carbapenemase gene-negative *Enterobacterales* (CgNE) infections were generally similar, except that CgPE cases were less likely to involve intra-abdominal infection than CgNE cases. Four species, namely *Klebsiella aerogenes*, *Enterobacter cloacae* complex (ECC), *K. pneumoniae*, and *E. coli*, accounted for approximately 80% of the reported pathogens. No isolates of *K. aerogenes*, the most reported species, were CgPE, and *K. pneumonia,* and *E. coli* were observed more frequently in CgPE cases than in CgNE cases. The predominant carbapenemase gene was *bla*_IMP_ (183, 93.4%), whereas few other carbapenemase genes were observed [*bla*_NDM_ and *bla*_KPC_ in eight (4.1%) and five cases (2.6%), respectively]. The all-cause 7-day mortality was 4.5% among 740 CRE cases with pathogen surveillance data, and the rate was higher for CgPE cases (6.1%) than for CgNE cases (3.9%), although the difference was not statistically significant.

**Table 1:** Baseline characteristics of cases with carbapenem-resistant *Enterobacterales* infection reported to the National Epidemiological Surveillance of Infectious Diseases

Characteristic	All reported cases	Cases with pathogen surveillance data	CgPE cases	CgNE cases	*P* value
	(*N* = 1681)	(*N* = 740)	(*N* = 196)	(*N* = 544)	
Age, median [IQR]	76 [67–83]	77 [68–84]	78 [69–85]	76 [67–84]	0.085
Male (N, %)	1045 (62.2%)	454 (61.4%)	112 (57.1%)	342 (62.9%)	0.171
Type of infection (N, %)					
Urinary tract infection	542 (32.2%)	244 (33.0%)	76 (38.8%)	168 (30.9%)	0.051
Bloodstream infection	491 (29.2%)	208 (28.1%)	64 (32.7%)	144 (26.5%)	0.115
Intra-abdominal infection	454 (27.0%)	207 (28.0%)	31 (15.8%)	176 (32.4%)	< 0.001
Respiratory tract infection	368 (21.9%)	171 (23.1%)	53 (27.0%)	118 (21.7%)	0.913
Bone and soft tissue infection	138 (8.2%)	61 (8.2%)	16 (8.2%)	45 (8.3%)	1.000
Others	16 (1.0%)	6 (0.8%)	1 (0.5%)	5 (0.9%)	1.000
Not specified	5 (0.3%)	2 (0.3%)	0 (0%)	2 (0.4%)	0
Multiple types of infection	305 (18.1%)	144 (19.5%)	40 (20.4%)	104 (19.1%)	0.752
Bacterial species (N, %)					
*Klebsiella aerogenes*	595 (35.4%)	241 (32.6%)	0 (0%)	241 (44.3%)	< 0.001
*Enterobacter cloacae* complex	487 (29.0%)	202 (27.3%)	60 (30.6%)	142 (26.1%)	0.226
*Klebsiella pneumoniae*	165 (9.8%)	86 (11.6%)	53 (27.0%)	33 (6.1%)	< 0.001
*Escherichia coli*	129 (7.7%)	68 (9.2%)	38 (19.4%)	30 (5.5%)	< 0.001
*Serratia marcescens*	71 (4.2%)	29 (3.9%)	4 (2.0%)	25 (4.6%)	0.135
Others	192 (11.4%)	97 (13.1%)	37 (18.9%)	60 (11.0%)	0.007
Not reported	48 (2.9%)	18 (2.4%)	4 (2.0%)	14 (2.6%)	0.793
Detected carbapenemase gene (N, %)					
*bla*_IMP_	–	183 (24.7%)	183 (93.4%)	–	–
*bla*_NDM_^a^	–	8 (1.1%)	8^a^ (4.1%)	–	–
*bla*_KPC_^b^	–	5 (0.7%)	5^b^ (2.6%)	–	–
*bla*_OXA-48-like_	–	0 (0%)	0 (0%)	–	–
Cases with reported date of death (N, %)	68 (4.0%)	33 (4.5%)	12 (6.1%)	21 (3.9%)	0.225

*IQR* interquartile range: *CgPE* carbapenemase gene-positive *Enterobacterales*: *CgNE* carbapenemase gene-negative *Enterobacterales*

^a^Bacterial species for *bla*_NDM_-positive isolates were *K. pneumoniae* (n = 5) and *E. coli* (*n* = 3).^b^All isolates carrying *bla*_KPC_ were *K. pneumoniae*

### Seven-day mortality risk analysis

Thirty-three (4.5%) of the 740 CRE cases under pathogen surveillance were fatal (Supplemental Table 1). Univariate and multivariate analyses (Table 2) revealed that bloodstream infections, respiratory tract infections, and *E. coli* infections were significant risk factors for 7-day mortality, whereas carbapenemase gene positivity was not significant [odds ratio (OR) = 1.62, 95% confidence interval (CI) = 0.78–3.37, *p* = 0.225].

**Table 2:** Seven-day mortality risk analysis among CRE cases with pathogen surveillance data (N = 740)

	CRE cases with pathogen surveillance data (N = 740)			
	Univariate analysis		Multivariate analysis	
Covariate	OR (95% CI)	P	aOR (95% CI)	P
Male	1.72 (0.79–3.75)	0.202	–	–
Age < 18 years	1.92 (0.43–8.51)	0.308	–	–
Age > 64 years	2.43 (0.73–8.06)	0.174	–	–
Urinary tract infection	0.44 (0.18–1.08)	0.087	–	–
Bloodstream infection	2.87 (1.42–5.8)	0.005	4.00 (1.88–8.52)	0.0003
Respiratory tract infection	2.94 (1.45–5.97)	0.005	4.71 (2.17–10.24)	< 0.0001
Intra-abdominal infection	1.3 (0.62–2.74)	0.552	–	–
Bone and soft tissue infection	0.71 (0.17–3.03)	1.000	–	–
*Escherichia coli*	4.12 (1.83–9.27)	0.002	5.17 (2.18–12.24)	0.0002
*Klebsiella aerogenes*	0.65 (0.29–1.47)	0.346	–	–
*Klebsiella pneumoniae*	0.75 (0.22–2.52)	1.000	–	–
*Enterobacter cloacae* complex	0.85 (0.38–1.91)	0.842	–	–
*Serratia marcescens*	2.62 (0.75–9.14)	0.134	–	–
Carbapenemase gene-positive *Enterobacterales*	1.62 (0.78–3.37)	0.225	–	–

*aOR* adjusted odds ratio: *OR* odds ratio: *CI* confidence interval

When the assessment specifically focused on determining the 7-day mortality risk among CgPE infections, positivity for the KPC/NDM-type carbapenemase gene was a significant independent risk factor compared to IMP-type carbapenemase (Table 3). Cases associated with the KPC/NDM-type carbapenemase gene most frequently manifested as bloodstream infection, which was identified as one of the significant risk factors in the 7-day mortality risk analysis for CRE. Conversely, urinary tract infection was the most reported manifestation in cases associated with IMP-type carbapenemase (Supplemental Table 2).

**Table 3:** Seven-day mortality risk analysis among CgPE cases (N = 196)

	Univariate analysis	
	OR (95% CI)	P
Male	1.05 (0.32–3.44)	1.000
Age < 18 years	2.3 (0.26–20.38)	0.402
Age > 64 years	1.81 (0.22–14.62)	1.000
Urinary tract infection	0.3 (0.06–1.4)	0.133
Bloodstream infection	3.12 (0.95–10.25)	0.061
Respiratory tract infection	2.91 (0.9–9.48)	0.09
Intra-abdominal infection	0.47 (0.06–3.75)	0.695
Bone and soft tissue infection	1.02 (0.8–8.48)	1.000
*Escherichia coli*	2.21 (0.63–7.75)	0.253
*Klebsiella pneumoniae*	0.52 (0.11–2.46)	0.519
*Enterobacter cloacae* complex	1.14 (0.33–3.95)	1.000
*Serratia marcescens*	5.48 (0.53–57.14)	0.225
*bla*_NDM,_ *bla*_KPC_	5.8 (1.36–24.82)	0.036

*OR* odds ratio, *CI* confidence interval

### Antimicrobial susceptibility

Among the 740 cases with pathogen surveillance data, 357 isolates from the same number of cases (357/740, 48.2%) underwent AST at NIID. The species distribution (*p* = 0.0776) and CgPE proportion (28.2% vs. 26.5% in pathogen surveillance, *p* = 0.562) of the 357 isolates did not differ significantly from those of the 740 isolates identified during pathogen surveillance.

**Fig. 1:**
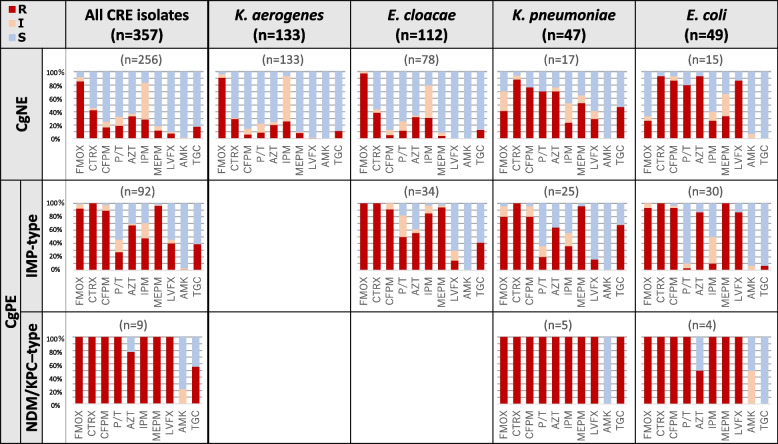
Antimicrobial susceptibility patterns of CRE isolates. Proportions of isolates resistant (R), intermediate (I), and susceptible (S) to the indicated antimicrobials as specified below the graphs, are represented by red, yellow, and blue bars. Antimicrobial abbreviations: FMOX (flomoxef), CTRX (ceftriaxone), CFPM (cefepime), P/T (piperacillin/tazobactam), AZT (aztreonam), IPM (imipenem), MEPM (meropenem), LVFX (levofloxacin), AMK (amikacin), TGC (tigecycline)

Figure 1 depicts the susceptibility to ten antimicrobials by species and the presence or absence of the carbapenemase gene. The proportion of multidrug resistance, defined as resistance to more than two of four commonly used broad-spectrum antimicrobials other than carbapenems in Japan (cefepime, piperacillin–tazobactam, levofloxacin, and amikacin), was 29.1% among the 357 CRE isolates, and the proportion was significantly higher in CgPE cases than in CgNE cases (65.4% vs. 14.8%, OR = 10.8; 95% CI = 6.33–18.48, *p* < 0.0001).

*K. aerogenes* and carbapenemase gene-negative ECC retained susceptibility to various types of antimicrobials, where the proportions of resistance to cefepime, piperacillin–tazobactam, levofloxacin, and amikacin were 5.7, 10.0, 0.5, and 0%, respectively, leading to a low proportion of multidrug resistance among all CgNE isolates. Conversely, *K. pneumoniae* and *E. coli* displayed no difference in the proportion of multidrug resistance regarding carbapenemase gene possession (*E. coli*: 86.7% for CgNE vs. 88.2% for CgPE, *p* = 1.000; *K. pneumoniae*: 64.7% for CgNE vs. 46.7% for CgPE, p = 1.000). Furthermore, *E. coli* exhibited remarkably high levofloxacin resistance. CgNE-*E. coli* and CgPE-*E. coli* displayed equally high levels of levofloxacin resistance (86.7 and 88.2%, respectively). Among the carbapenemase types, NDM/KPC-type CgPE exhibited a distinctively higher proportion of multidrug resistance than IMP-type CgPE (100% vs. 62.0%, *p* = 0.025). All NDM/KPC-type CgPE isolates were resistant to cefepime, piperacillin–tazobactam, and levofloxacin. By contrast, 89, 27, and 40% of IMP-type CgPE isolates were resistant to cefepime, piperacillin–tazobactam, and levofloxacin, respectively.

Regarding the mortality risk associated with multidrug resistance, CRE isolates simultaneously resistant to three broad-spectrum antimicrobials, namely cefepime, piperacillin–tazobactam, and levofloxacin, carried a higher risk of 7-day mortality than isolates not resistant to these antimicrobials (13.8% vs. 3.7%, OR = 4.22. 95% CI = 1.27–14.03, *p* = 0.0593). However, the observed difference was not significant, possibly because of a lack of statistical power attributable to the small number of fatal cases.

### Comparison of the two CRE criteria groups

To compare the clinical characteristics of CRE cases identified by imipenem resistance and meropenem resistance, two groups were defined: the “IPM criteria-only group,” which fulfilled only the IPM criteria and not the MEPM criteria, and the “MEPM criteria group,” which met the MEPM criteria regardless of imipenem resistance. Among the 357 isolates, 315 (88.2%) met the CRE definition based on AST results at the NIID. Among them, 169 isolates (53.7%) were categorised into the IPM criteria-only group, and 146 (46.3%) belonged to the MEPM criteria group. The majority (71.2% 104/146) of the isolates in the MEPM criteria group simultaneously fulfilled the IPM criteria, indicating resistance to both imipenem and meropenem (Table 4).

**Table 4:** IPM-criteria-only group and MEPM-criteria group: Comparison among the isolates satisfying Japanese CRE criteria at national central laboratory (*N* = 315)

N (%)	IPMcriteria-only group ***N*** = 169	MEPM criteria group ***N*** = 146	***P***-value
IPM criteria fulfilled	169 (100%)	104 (71.2%)	–
*Klebsiella aerogenes*	109 (64.5%)	13 (8.9%)	< 0.0001
*Enterobacter cloacae* complex	55 (32.5%)	40 (27.4%)	0.328
*Klebsiella pneumoniae*	0 (0%)	40 (27.4%)	< 0.0001
*Escherichia coli*	1 (0.6%)	44 (30.1%)	< 0.0001
*Serratia marcescens*	4 (2.4%)	9 (6.2%)	0.153
CgPE	0 (0%)	99 (67.8%)	< 0.0001
Multidrug resistance	2 (1.2%)	96 (65.8%)	< 0.0001
7-day-mortality	3 (1.8%)	10 (6.9%)	0.0428
US-CDC CRE reporting criteria^a^ fulfilled	46 (27.2%)	146 (100%)	< 0.0001
Meropenem MIC≥4	0 (0%)	128 (87.7%)	< 0.0001
Imipenem MIC≥4	41 (24.3%)	85 (58.2%)	< 0.0001
Ertapenem MIC≥2	10 (5.9%)	146 (100%)	< 0.0001

IPMcriteria-only group = Isolates meeting the criteria only for imipenem MIC≥2 and cefmetazole MIC≥64

MEPM criteria group = Isolates meeting the criteria for meropenem MIC≥2, irrespective of imipenem resistance

^a^ US CDC-CRE criteria definition: (a) Resistant to any carbapenem antimicrobial (i.e., minimum inhibitory concentrations of ≥4 mcg/ml for doripenem, meropenem, or imipenem OR ≥ 2 mcg/ml for ertapenem); (b) In addition: (i) For bacteria that have intrinsic imipenem nonsusceptibility (i.e., *Morganella morganii*, *Proteus* spp., *Providencia* spp.), resistance to carbapenems other than imipenem is required, OR (ii) Documented to produce carbapenemase

The species distribution differed between the IPM criteria-only and MEPM criteria groups. *K. aerogenes* comprised more than 60% of isolates in the IPM criteria-only group, whereas *E. coli* and *K. pneumoniae* accounted for nearly 60% of isolates in the MEPM criteria group. Notably, CgPE isolates were not found in the IPM criteria-only group, whereas 67.8% of the isolates in the MEPM criteria group were CgPE. The proportion of multidrug resistance was low in the IPMcriteria-only group (1.2%), 91.1% of the isolates in the IPM criteria-only group were susceptible to all four antimicrobials. Seven-day mortality was also significantly lower among in the IPMcriteria-only group than in the MEPM criteria group (1.8% vs. 6.9%, *p* = 0.0428).

In addition, 61.0% (192/315) of the isolates satisfied the CRE definition of the US CDC. The CRE definition of US CDC had two-fold higher thresholds for the MICs of meropenem and imipenem than the Japanese CRE definition. Among isolates in the IPMcriteria-only group, 24.3% fulfilled the US-CDC CRE criteria for imipenem, and 95.1% (39/41) of them remained susceptible to cefepime, piperacillin–tazobactam, levofloxacin, and amikacin. Meanwhile, all isolates in the MEPM criteria group fulfilled the US CDC CRE criteria. This was because all 18 isolates in the MEPM criteria group, which had a meropenem MIC of 2 mg/L, exhibited an MIC of 2 mg/L for ertapenem. One of these 18 isolates was an IMP-type carbapenemase producer.

## Discussion

In Japan, among the reported CRE cases, the dominant species were *K. aerogenes* and ECC, followed by *K. pneumoniae* and *E. coli*. The proportion of CgPE isolates was 26.5%, and the 7-day mortality was 4.5%. Both of these figures were lower than those reported in other countries [4, 14, 15], but they were consistent with previous domestic studies that used the same CRE definition [16, 17]. However, one of these previous studies reported higher mortality rates than the present study. Specifically, Oka et al. reported a 28-day mortality rate of 14.6% [17]. This difference might be attributable to the study populations and longer observational periods, as their study was conducted at university hospitals in which patients with more complex clinical backgrounds were more prevalent, whereas our study, which included cases from primary hospitals.

One of the reasons for prioritizing CPE among CRE isolates is that CPE is associated with a significantly higher risk of mortality than infection caused by non-CPE [18, 19]. However, recent studies revealed no significant differences in clinical outcomes between CPE and non-CPE infections [14, 16, 17, 20, 21]. Our analysis also did not conclude that CgPE posed a greater risk of mortality. Conversely, it revealed a higher proportion of multidrug resistance among CgPE isolates than in among CgNE isolates. Furthermore, CRE isolates exhibiting multidrug resistance carried a notably high risk of 7-day mortality. These findings that multidrug resistance, rather than the mere possession of carbapenemase genes, exerts a stronger impact on clinical outcomes. In other words, the previously reported causal association between CPE infection and mortality might be confounded by the presence of multidrug resistance. Recently, novel antimicrobials targeting CPE have been introduced in clinical settings, including new β-lactam–β-lactamase inhibitor combinations (e.g., imipenem–relebactam and ceftazidime–avibactam) and cefiderocol. These antimicrobials can alter the clinical outcomes of CRE infections, particularly those caused by CPE.

The significant difference in the proportion of multidrug resistance between CgPE and CgNE isolates in this study might be attributable to the large proportion (82.4%, 211/256, Fig. 1) of *K. aerogenes* and ECC isolates in CgNE. These isolates remained susceptible to various antimicrobials, thereby reducing the overall proportion of multidrug resistance among CgNE isolates. The reason for high susceptibility to various antimicrobials but not to imipenem and cefmetazole in *K. aerogenes* and CgNE-ECC might be explained by the presence of chromosomal AmpC β-lactamase production combined with altered membrane permeability, which can elevate the MIC of imipenem even though they are not exogenously acquired resistance mechanisms but can elevate the MIC of imipenem [22–24].

In the analysis of mortality risk, cases other than *K. aerogenes* and ECC infections, particularly those involving *E. coli* and *K. pneumoniae*, accounted for half of the fatal cases and likely influenced the risk analysis. It is important to recognize that the proportion of multidrug resistance in *E. coli* and *K. pneumoniae* did not differ between CgPE and CgNE. In particular, the proportion of multidrug resistance in *E. coli* exceeded 85% regardless of the presence or absence of carbapenemase genes. This could be one of the explanations for the novel finding that *E. coli* infection itself increased the risk of early mortality among cases with CRE infection.

Unlike *K. aerogenes* and ECC, CgNE-*E. coli* and CgNE-*K. pneumoniae* typically exhibit elevated MICs for carbapenems only when they acquire exogenous β-lactamase genes, such as plasmid-mediated ESBLs and AmpC β-lactamase genes, alongside altered membrane permeability [25, 26]. This propensity for co-acquiring other exogenous resistance genes, such as aminoglycoside resistance genes, reflects the scenario for CPE, which acquires exogenous carbapenemase genes and other resistance genes and consequently develops multidrug resistance. In addition, the distinct high rate of resistance to levofloxacin, one of the commonly used antimicrobials in Japan [27], observed in *E. coli* isolates might also contribute to multidrug resistance and higher mortality rates because of empirical treatment failure.

IMP-type carbapenemase is a domestically prevalent carbapenemase in Japan, whereas NDM/KPC-type carbapenemase was more associated with cases with a history of travel abroad [28]. Within the CgPE cases analyzed, NDM/KPC-CgPE increased the risk of mortality compared to IMP-type CgPE. This finding might also be attributable to the higher prevalence of multidrug resistance in NDM/KPC-CgPE than in IMP-type CgPE.

Despite slight variations in mortality data among domestic studies, the characteristics of CRE cases in Japan, including the high proportion of *K. aerogenes* and ECC, relatively lower proportion of CgPE, and the low mortality, were distinctive. We assumed that these features in Japan might be explained by the specific Japanese CRE definition, particularly among cases that met only the IPM criteria, forming the IPMcriteria-only group in this study. This group comprised 97% *K. aerogenes* and ECC isolates, a single *E. coli* isolate, and no *K. pneumoniae* isolates, and no isolates were CgPE. Furthermore, this group exhibited a low proportion of multidrug resistance (1.2%). Of the isolates in the IPMcriteria-only group, which remained susceptible to meropenem, 91% were also susceptible to cefepime, piperacillin–tazobactam, levofloxacin, and amikacin, meaning that sufficient treatment options were available, which could explain the low mortality of 1.8% in this group.

In the IPMcriteria-only group, 24.3% of the patients exhibited an imipenem MIC of ≥4 mg/L, which aligns with the cutoff used in the US-CDC CRE criteria [11]. These isolates also remained susceptible to other antimicrobials used to treat CRE infections. Regardless of its MIC cutoff, imipenem is an inappropriate reference drug for the definition of CRE surveillance.

This study had several limitations. First, the NESID does not provide any definition or guideline for the clinical diagnosis of CRE infection, and all diagnoses are based on the clinician’s discretion. Second, the clinical aspects of the cases, such as underlying diseases and treatment courses, were unavailable, and such information is crucial for evaluating mortality risks in real-world settings. Furthermore, because the mortality risk was analyzed under the limitation of surveillance data, which were collected within 7 days of diagnosis, a longer observation period would provide a more precise view of mortality. Third, the number of isolates tested for antimicrobial susceptibility was limited. Finally, the findings of this study were derived from an analysis of CRE cases in Japan, where IMP-type carbapenemase is dominant. Further study in regions with different dominant carbapenemase gene profiles is required to evaluate the significance of imipenem resistance in defining CRE.

## Conclusion

In summary, in cases in the IPMcriteria-only group, in which CRE was identified solely based on imipenem resistance, the isolates were less likely to be CgPE. Furthermore, and more importantly, these isolates were less likely to exhibit multidrug resistance, indicating that existing antimicrobials are more likely to be effective, potentially resulting in a lower clinical and public health burden. To preserve newly developed antimicrobials such as ceftazidime–avibactam, meropenem–vaborbactam, imipenem–cilastatin–relebactam, and cefiderocol and to efficiently allocate limited healthcare and public health resources for infection control, it is recommended that CRE is defined solely using the MEPM criteria. This approach will help accurately understand the disease burden of CRE infection and the challenges it presents in clinical management and infection control.

### Supplementary Information

**Additional file 1. **Supplementary material 1.

## Data Availability

NESID reports are available in the public domain on the NIID website (https://www.niid.go.jp/niid/ja/) as the Infectious Diseases Weekly Report and Infectious Agents Surveillance Report.

## Abbreviations

AST: Antimicrobial susceptibility test
CLSI: Clinical and Laboratory Standards Institute
CgNE: Carbapenemase gene-negative *Enterobacterales*
CgPE: Carbapenemase gene-positive *Enterobacterales*
CRE: Carbapenem-resistant *Enterobacterales*
CPE: Carbapenemase-producing *Enterobacterales*
ECC: *Enterobacter cloacae* complex
EDTA: Ethylenediaminetetraacetic acid
ESBL: Extended-spectrum β-lactamase
IPM: Imipenem
MEPM: Meropenem
MICs: Minimum inhibitory concentrations
NESID: National Epidemiological Surveillance of Infectious Diseases
PHI: Public Health Institute
US-CDC: Centers for Disease Control and Prevention of United States
