# Supplementary material for: The importance of meropenem resistance, rather than imipenem resistance, in defining carbapenem-resistant Enterobacterales for public health surveillance: an analysis of national population-based surveillance
Source: BMC Infect Dis. 2024 Feb 15;24:209. doi: 10.1186/s12879-024-09107-4 (PMC10870673; doi:10.1186/s12879-024-09107-4)
Supplement: Supplementary file 1 — Additional file 1. Supplementary material 1. [file 12879_2024_9107_MOESM1_ESM.docx]

**Supplemental Table 1. Demographics of cases of fatality within 7 days of clinical diagnosis among carbapenem-resistant *Enterobacterales* infection cases according to the National Epidemiological Surveillance of Infectious Diseases (N=33)**

|  | Cases with pathogen surveillance data | CgPE cases | CgNE cases |
| --- | --- | --- | --- |
| Cases of fatality within 7 days of clinical diagnosis (N) | 33 | 12 | 21 |
| Male (N, %) | 24 (72.7) | 7 (58.3) | 17 (80.9) |
| Age <18 years | 2 (6.1) | 1 (8.3) | 1 (4.8) |
| Age >64 years | 30 (90.9) | 11 (91.7) | 19 (90.5) |
|  |  |  |  |
| Type of infection (N, %) |  |  |  |
| Urinary tract infection | 6 (18.2) | 2 (16.7) | 4 (19.0) |
| Bloodstream infection | 17 (51.5) | 7 (58.3) | 10 (47.6) |
| Intra-abdominal infection | 11 (33.3) | 1 (8.3) | 10 (47.6) |
| Respiratory tract infection | 15 (45.5) | 6 (50.0) | 9 (42.9) |
| Bone and soft tissue infection | 2 (6.1) | 1 (8.3) | 1 (4.8) |
|  |  |  |  |
| Multiple types of infection | 15 (45.5) | 5 (41.7) | 10 (47.6) |
|  |  |  |  |
| Reported bacterial species (N, %) |  |  |  |
| *Klebsiella aerogenes* | 8 (24.2) | 0 (0.0) | 8 (38.1) |
| *Enterobacter cloacae* complex | 8 (24.2) | 4 (33.3) | 4 (19.0) |
| *Klebsiella pneumoniae* | 3 (9.1) | 2 (16.7) | 1 (4.8) |
| *Escherichia coli* | 9 (27.3) | 4 (33.3) | 5 (23.8) |
| *Serratia marcescens* | 3 (9.1) | 1 (8.3) | 2 (9.5) |
| *Klebsiella oxytoca* | 2 (6.1) | 1 (8.3) | 1 (4.8) |
|  |  |  |  |
| Detected carbapenemase gene (N, %) |  |  |  |
| *bla*_IMP_ |  | 9 (75.0) |  |
| *bla*_NDM,_ *bla*_KPC_ |  | 3 (25.0) |  |

CgNE, carbapenemase gene-negative *Enterobacterales*;

CgPE, carbapenemase gene-positive *Enterobacterales*

**Supplemental Table 2. Comparison of baseline characteristics between cases with IMP-type and KPC/NDM-type carbapenemase-producing *Enterobacterales* among CgPE cases (N=196)**

| Characteristic | IMP cases | KPC/NDM cases | P value |
| --- | --- | --- | --- |
|  | (N = 183; 93.4%) | (N = 13; 6.6%) |  |
| Age (median, IQR) | 78 [69–86] | 76 [47.5-81] | 0.217 |
| Male (N, %) | 103 (56.3%) | 9 (69.2%) | 0.403 |
|  |  |  |  |
| Type of infection (N, %) |  |  |  |
| Urinary tract infection | 72 (39.3%) | 4 (30.8%) | 0.770 |
| Bloodstream infection | 59 (32.2%) | 5 (38.5%) | 0.761 |
| Intra-abdominal infection | 30 (16.4%) | 1 (7.7%) | 0.696 |
| Respiratory tract infection | 49 (26.8%) | 4 (30.8%) | 0.752 |
| Bone and soft tissue infection | 14 (7.7%) | 2 (15.4%) | 0.287 |
| Others | 1 (0.5%) | 0 (0%) | 1.000 |
| Not specified | 0 (0%) | 0 (0%) | - |
|  |  |  |  |
| Multiple types of infection | 37 (20.2%) | 3 (23.1%) | 0.731 |
| Bloodstream infection with intra-abdominal infection | 10 (5.5%) | 1 (7.7%) | 0.540 |
| Bloodstream infection with urinary tract infection | 9 (4.9%) | 1 (7.7%) | 0.505 |
| Bloodstream infection with respiratory tract infection | 9 (4.9%) | 0 (0%) | 1.000 |
| Others | 9 (4.9%) | 1 (7.7%) | 0.505 |
|  |  |  |  |
| Reported bacterial species (N, %) |  |  |  |
| *Klebsiella aerogenes* | 0 (0%) | 0 (0%) | - |
| *Enterobacter cloacae* complex | 60 (32.8%) | 0 (0%) | 0.011 |
| *Klebsiella pneumoniae* | 45 (24.6%) | 8 (61.5%) | 0.007 |
| *Escherichia coli* | 34 (18.6%) | 4 (30.8%) | 0.284 |
| *Serratia marcescens* | 4 (2.2%) | 0 (0%) | 1.000 |
| Others | 37 (20.2%) | 0 (0%) | 0.134 |
| Not reported | 3 (1.6%) | 1 (7.7%) | 0.242 |
|  |  |  |  |
| Cases with reported date of death (N, %) | 9 (4.9%) | 3 (23.1%) | 0.036 |

IQR, interquartile range; CgPE, carbapenemase gene-positive *Enterobacterales*
